# Hemorrhagic Cystitis Requiring Bladder Irrigation is Associated with Poor Mortality in Hospitalized Stem Cell Transplant Patients

**DOI:** 10.1590/S1677-5538.IBJU.2014.0655

**Published:** 2015

**Authors:** Valary T. Raup, Aaron M. Potretzke, Brandon J. Manley, John A. Brockman, Sam B. Bhayani

**Affiliations:** 1 Division of Urology, Washington University School of Medicine, Washington, DC, USA

**Keywords:** Bone Marrow Transplantation, Stem Cell Transplantation, Cystitis, Hematuria

## Abstract

**Purpose:**

To evaluate the overall prognosis of post-stem cell transplant inpatients who required continuous bladder irrigation (CBI) for hematuria.

**Materials and Methods:**

We performed a retrospective analysis of adult stem cell transplant recipients who received CBI for de novo hemorrhagic cystitis as inpatients on the bone marrow transplant service at Washington University from 2011-2013. Patients who had a history of genitourinary malignancy and/or recent surgical urologic intervention were excluded. Multiple variables were examined for association with death.

**Results:**

Thirty-three patients met our inclusion criteria, with a mean age of 48 years (23-65). Common malignancies included acute myelogenous leukemia (17/33, 57%), acute lymphocytic leukemia (3/33, 10%), and peripheral T cell lymphoma (3/33, 10%). Median time from stem cell transplant to need for CBI was 2.5 months (0 days-6.6 years). All patients had previously undergone chemotherapy (33/33, 100%) and 14 had undergone prior radiation therapy (14/33, 42%). Twenty-eight patients had an infectious disease (28/33, 85%), most commonly BK viremia (19/33, 58%), cytomegalovirus viremia (17/33, 51%), and bacterial urinary tract infection (8/33, 24%). Twenty-two patients expired during the same admission as CBI treatment (22/33 or 67% of total patients, 22/28 or 79% of deaths), with a 30-day mortality of 52% and a 90-day mortality of 73% from the start of CBI.

**Conclusions:**

Hemorrhagic cystitis requiring CBI is a symptom of severe systemic disease in stem cell transplant patients. The need for CBI administration may be a marker for mortality risk from a variety of systemic insults, rather than directly attributable to the hematuria.

## INTRODUCTION

Hemorrhagic cystitis is a significant cause of morbidity in immunocompromised patients on the Bone Marrow Transplant (BMT) service, occurring in up to 30% of hematopoietic stem cell transplant recipients (1–3). The most prevalent causes of hemorrhage include toxic effects of chemotherapy, infection, and radiation cystitis (2). Cyclophosphamide and busulfan are the most common chemotherapeutic agents causing hemorrhagic cystitis, while polyoma BK virus, adenovirus, and cytomegalovirus are the most common infectious agents (4, 5). Polyoma BK viremia, specifically, has been shown to be correlated with increased severity of hemorrhagic cystitis but not with increased mortality (6). Other reported predisposing factors to the development of hemorrhagic cystitis include thrombocytopenia, coagulopathy, and possibly graft-versus-host disease (GVHD) (7).

Often urologic consultation is obtained on these patients when there is a need for continuous bladder irrigation (CBI). The primary goals of this study were to characterize the patients requiring CBI for hemorrhagic cystitis after hematopoietic stem cell transplant and assess all-cause mortality of this cohort.

## MATERIALS AND METHODS

A retrospective review was performed of 33 adult patients who received CBI for hemorrhagic cystitis as inpatients on the BMT service at Washington University between 2011-2013. Data collection began after receiving Institutional Review Board approval. Only patients who had undergone prior hematopoietic stem cell transplant were included in the study, and patients who had a history of genitourinary malignancy and/or had undergone a recent surgical urologic intervention were excluded.

Pre-CBI complete blood count values were collected < 24 hours prior to the start of CBI treatment. Infectious diseases were identified with blood culture, urine culture, or quantitative viral polymerase chain reaction analysis, as appropriate. CBI treatment was provided through a triple lumen urinary catheter. All patients initially received intravesical irrigations of saline solution. Clinical variables were retrospectively reviewed, and outcomes were assessed to see if hemorrhagic cystitis was associated with prognosis or systemic disease.

Student's t-test was used to compare continuous, normally distributed variables, and Welch's t-test was used to compare continuous, non-normally distributed variables. All statistical analyses were two-sided using a significance of p≤0.05.

## RESULTS

### Patient Characteristics

Mean age of the study population was 48 years (range 23-65), with a median Charleston Comorbidity Index (CCI) of 2 (0-6). Thirty patients had a hematologic malignancy (30/33, 91%), 2/33 patients had myelodysplastic syndrome (MDS) (6%), and 1/33 patient had aplastic anemia (3%). Malignancies included acute myelogenous leukemia (AML) (17/33, 57%), acute lymphoblastic leukemia (ALL) (3/33, 10%), peripheral T cell lymphoma (3/33, 10%), diffuse large B cell lymphoma (2/33, 7%), Hodgkin's lymphoma (2/33, 7%), multiple myeloma (1/33, 3%), Mantle cell lymphoma (1/33, 3%), and Sézary syndrome (1/33, 3%). Details of the patients’ characteristics and relation to mortality can be found in Table-1. As expected, malignancy demonstrated a significant association with mortality (p=0.015).

**Table 1:** patient characteristics and malignancy subtype as risk factors for 90-day mortality

Clinical Characteristics		Total (n=33)	Deceased at 90 days (n=24)	P-value
Average Age (in years)		48	49	
Median CCI		2	2	1.000
Malignancy		30	24	0.015
	AML	17	14	0.259
	MM	1	1	1.000
	ALL	3	3	0.545
	PTCL	3	2	1.000
	DLBCL	2	1	0.477
	HL	2	2	1.000
	ML	1	1	1.000
	SS	1	0	0.273
MDS		2	0	0.068
Aplastic Anemia		1	0	0.273

**CCI** = Charleston Comorbidity Index; **AML** = acute myelogenous leukemia; **MM** = multiple myeloma; **ALL** = acute lymphoblastic leukemia; **PTCL** = peripheral T cell lymphoma; **DLBCL** = diffuse large B cell lymphoma; **HL** = Hodgkin's lymphoma; **ML** = Mantle cell lymphoma; **SS** = Sezary syndrome.

All patients had received a prior hematopoietic stem cell transplant, with a median time from transplant to need for CBI of 2.5 months (range: 0 days-6.6 years). All 33 patients also had previously undergone chemotherapy, with the most prevalent agents including cyclophosphamide (25), cytarabine (21), busulfan (13), vincristine (11), idarubicin (10), and etoposide (10). Fourteen patients had undergone prior radiation therapy (14/33, 42%), none of which had treatment directed to the pelvis. Details of treatment characteristics and relation to mortality can be found in Table-2. Radiation or chemotherapy alone were not independent risk factors for mortality, but obviously may be correlated with stem cell transplant, therefore they cannot be exclusively separated. No specific prior oncologic treatment was uniquely significant over other treatments.

**Table 2:** prior oncological treatments as risk factors for 90-day mortality.

Treatment		Total	Deceased (at 90 days)	P-value
**Radiation**		14	9	0.442
**Chemotherapy**				
	Cyclophosphamide	25	17	0.394
	Cytarabine	21	17	0.230
	Idarubicin	10	8	0.686
	Etoposide	10	7	1.000
	Busulfan	13	10	1.000
	Vincristine	11	9	0.681

Twenty-eight patients were found to have an infectious disease (28/33, 85%), most commonly BK viremia (19/33, 58%), cytomegalovirus viremia (17/33, 51%), and bacterial urinary tract infection (UTI) (8/33, 24%). Twenty-three patients had pre-existing GVHD (23/33, 70%), frequently of the gastrointestinal (GI) tract (16/33, 48%), skin (6/33, 18%), or liver (5/33, 15%). Median pre-CBI complete blood count values showed patients to be leukopenic at 1.9 x 10^3^/μL, anemic at 9.4g/dL and thrombocytopenic at 18 x 10^3^/μL. Details of infectious diseases and GVHD and their association with mortality can be found in Table-3.

**Table 3:** Additional possible risk factors of mortality.

Risk Factor		Total	Deceased (at 90 days)	P-value
**Infectious Disease**		28	22	0.111
	BK Virus	19	14	1.000
	CMV	17	12	1.000
	VRE UTI	6	4	1.000
	Klebsiella UTI	2	1	0.477
	Enterobacter UTI	1	1	1.000
**GVHD**		23	16	0.547
	Gastrointestinal	16	13	0.111
	Skin	6	3	0.653
	Liver	5	5	0.143

**CMV** = cytomegalovirus; **VRE** = vancomycin-resistant enterococci; **UTI** = urinary tract infection; **GVHD** = graft-versus-host disease

Two patients eventually required instillation of 1% alum solution to control hemorrhage (2/33, 6%), and 6 patients required cystoscopy with clot evacuation after initiation of CBI (6/33, 18%) for removal of recurrent clots. Neither need for instillation of alum solution nor need for cystoscopy with clot evacuation was shown to be associated with changes in 30- or 90-day mortality (30-day p=0.21, 0.40, 90-day p=0.47, 1.00).

### Descriptors of Mortality Analysis

Twenty-eight patients were deceased at the time of last follow-up (28/33, 85%). Average age at death was 49 years, with median time to death from initiation of CBI of 22 days (0-804 days). Of these patients, twenty-two patients died during the same admission of CBI treatment (22/28 or 79% of deaths, 22/33 or 67% of total patients). Thirty-day mortality was found to be 52% (17/33), and 90-day mortality was 73% (24/33).

We assessed factors possibly related to mortality in our cohort. Nearly all of the patients who were deceased at the time of last follow-up died from cancer related causes and subsequent multi-organ failure (27/28, 96%). Neither mean age nor median CCI at time of admission were shown to be associated with increased 30- or 90-day mortality. Treatment with chemotherapy in general or any specific agent was not shown to be associated with mortality. Similarly, radiation was not associated with increased mortality. Infectious diseases, pre-existing GVHD, neutropenia, anemia, and thrombocytopenia were also not shown to be specifically associated with increased mortality.

## DISCUSSION

Our findings demonstrate that hemorrhagic cystitis requiring CBI in patients having undergone stem cell transplant is associated with a high 30-day and 90-day mortality rate. A previous study from our institution showed a 90-day mortality rate of 12% after stem cell transplant (8). The present study, however, demonstrates a markedly higher mortality rate in this cohort with hemorrhagic cystitis requiring CBI, the 90-day mortality rate was 73%. It is important to note that this is an association, but not an assertion of cause. In our best assessment, this cohort's development of hemorrhagic cystitis seems to be part of an overall picture of severe medical illness, rather than a specific cause of mortality. Hence, hemorrhagic cystitis requiring CBI is likely a urological marker of severe systemic insults within this population. Hematuria was not the cause of death in these patients, but rather was just another marker for severe systemic disease.

Hemorrhagic cystitis is a significant cause of morbidity in stem cell transplant patients, with most common etiologies including toxic effects of chemotherapy, infection, and radiation cystitis. However, these patients regularly have many predisposing factors, thereby causality is difficult to assign. The statistical challenge of demonstrating significant relationships is compounded by the relatively small cohort size and overall high mortality risk across the entire cohort, thereby making it unlikely that any patient factor would be more significant than others (Tables 1–3). For example, cyclophosphamide has been shown to be the most common chemotherapeutic agent causing hemorrhagic cystitis in the literature (4, 9, 10). Most of the patients (88%) who received chemotherapy also had other risk factors for development of hematuria such as viruria, bacteria, or radiation, thus cause cannot be clearly identified. This further supports the idea that hemorrhagic cystitis is a symptom of severe systemic disease rather than a causative mortality factor.

In our study, more than half (67%) of the patients died during the same admission in which occurred CBI. The overall 30-day mortality was 52%, and 90-day mortality was 73%. Padilla-Fernandez et al. found a slightly lower overall mortality rate of 51.14% in a population of 52 post-hematopoietic stem cell transplant patients with hemorrhagic cystitis, although the authors did not specifically examine patients with severe enough hemorrhagic cystitis to merit CBI (11). In their study, hemorrhagic cystitis was also not found to be the cause of death in any patient. Nevertheless, this consistently high mortality risk should concern urologists in planning overall treatment and care of these patients. Given that no patients died directly from hemorrhage, and additional interventions were not found to impact mortality, perhaps treatment plans should be made with paramount consideration of patient comfort. Unless a patient is actively exsanguinating due to the severity of their hematuria, care may be limited to CBI, and clinicians should use this high mortality rate to more accurately advise families on prognosis.

Under more typical circumstances, treatments of hemorrhagic cystitis require the patient to be free of bladder clots, which can be achieved with aggressive manual irrigation or cystoscopy with clot evacuation and fulguration. Once the bladder is clear, continuous bladder irrigation is initiated to prevent further clot development and retention. Mild hemorrhages can be treated with sodium chloride instillation, while more severe cases require instillation of aminocaproic acid, alum, silver nitrate, phenol, or formaldehyde (12). In refractory cases, hyperbaric oxygen therapy or embolization of the internal iliac arteries can be employed (13, 14). Surgical options such as urinary diversion or cystectomy are an option if all other therapies are unsuccessful (15). Fibrin glue therapy is a novel technique that has been recently described by Tirindelli et al., who showed that this treatment increased the 6-month probability of survival when used in post-allogenic hematopoietic stem cell transplant patients (16).

Our study has several limitations. This project is both retrospective and hypothesis generating, thus larger-scale prospective studies are necessary to investigate possible associations between hemorrhagic cystitis, predisposing factors, and increased mortality. Nevertheless, a randomized or prospective study may not be practical or possible in this population. In addition, a matched cohort study comparing the mortality rates between similar patients with and without hemorrhagic cystitis would be necessary to truly elucidate a statistically significant risk of mortality in post-stem cell transplant patients with hemorrhagic cystitis.

## CONCLUSIONS

Hemorrhagic cystitis requiring CBI is an indicator of severe systemic disease in patients who underwent previous stem cell transplantation via either peripheral blood or BMT. The need for CBI administration may portend an increased risk of mortality for hospitalized stem cell transplant patients. The urologist has a unique perspective when receiving a consultation for CBI in a BMT patient. Counseling of the patient, family, and other providers is paramount. Further studies and improved strategies for management of these patients are needed.

## ABBREVIATIONS

CBI =: continuous bladder irrigation
BMT =: bone marrow transplant
AML =: acute myelogenous leukemia
ALL =: acute lymphoblastic leukemia
UTI =: urinary tract infection
GVHD =: graft-versus-host disease
MDS =: myelodysplastic syndrome
CCI =: Charleston Comorbidity Index
MM =: multiple myeloma
PTCL =: peripheral T cell lymphoma
DLBCL =: diffuse large B cell lymphoma
HL =: Hodgkin's lymphoma
ML =: Mantle cell lymphoma
SS =: Sézary syndrome
CMV =: cytomegalovirus
GI =: gastrointestinal
VRE =: vancomycin-resistant enterococcus

## References

[1] Seber A, Shu XO, Defor T, Sencer S, Ramsay N (1999). Risk factors for severe hemorrhagic cystitis following BMT. Bone Marrow Transplant.

[2] Sencer SF, Haake RJ, Weisdorf DJ (1993). Hemorrhagic cystitis after bone marrow transplantation. Risk factors and complications. Transplantation.

[3] Cesaro S, Brugiolo A, Faraci M, Uderzo C, Rondelli R, Favre C (2003). Incidence and treatment of hemorrhagic cystitis in children given hematopoietic stem cell transplantation: a survey from the Italian association of pediatric hematology oncology-bone marrow transplantation group. Bone Marrow Transplant.

[4] Brugieres L, Hartmann O, Travagli JP, Benhamou E, Pico JL, Valteau D (1989). Hemorrhagic cystitis following high-dose chemotherapy and bone marrow transplantation in children with malignancies: incidence, clinical course, and outcome. J Clin Oncol.

[5] Gorczynska E, Turkiewicz D, Rybka K, Toporski J, Kalwak K, Dyla A (2005). Incidence, clinical outcome, and management of virus-induced hemorrhagic cystitis in children and adolescents after allogeneic hematopoietic cell transplantation. Biol Blood Marrow Transplant.

[6] Gilis L, Morisset S, Billaud G, Ducastelle-Leprêtre S, Labussière-Wallet H, Nicolini FE (2014). High burden of BK virus-associated hemorrhagic cystitis in patients undergoing allogeneic hematopoietic stem cell transplantation. Bone Marrow Transplant.

[7] Silva Lde P, Patah PA, Saliba RM, Szewczyk NA, Gilman L, Neumann J (2010). Hemorrhagic cystitis after allogeneic hematopoietic stem cell transplants is the complex result of BK virus infection, preparative regimen intensity and donor type. Haematologica.

[8] Brown RA, Adkins D, Khoury H, Vij R, Goodnough LT, Shenoy S (1999). Long-term follow-up of high-risk allogeneic peripheral-blood stem-cell transplant recipients: graftversus-host disease and transplant-related mortality. J Clin Oncol.

[9] Cox PJ (1979). Cyclophosphamide cystitis–identification of acrolein as the causative agent. Biochem Pharmacol.

[10] Pode D, Perlberg S, Steiner D (1983). Busulfan-induced hemorrhagic cystitis. J Urol.

[11] Padilla-Fernandez B, Bastida-Bermejo JM, Virseda-Rodriguez AJ, Labrador-Gomez J, Caballero-Barrigon D, Silva-Abuin JM (2014). Hemorrhagic cytitis after bone marrow transplantation. Arch Esp Urol.

[12] Alesawi AM, El-Hakim A, Zorn KC, Saad F (2014). Radiation-induced hemorrhagic cystitis. Curr Opin Support Palliat Care.

[13] Giuliani L, Carmignani G, Belgrano E, Puppo P (1979). Gelatin foam and isobutyl-2-cyanoacrylate in the treatment of lifethreatening bladder haemorrhage by selective transcatheter embolisation of the internal iliac arteries. Br J Urol.

[14] Oscarsson N, Arnell P, Lodding P, Ricksten SE, Seeman-Lodding H (2013). Hyperbaric oxygen treatment in radiationinduced cystitis and proctitis: a prospective cohort study on patient-perceived quality of recovery. Int J Radiat Oncol Biol Phys.

[15] deVries CR, Freiha FS (1990). Hemorrhagic cystitis: a review. J Urol.

[16] Tirindelli MC, Flammia GP, Bove P, Cerretti R, Cudillo L, De Angelis G (2014). Fibrin glue therapy for severe hemorrhagic cystitis after allogeneic hematopoietic stem cell transplantation. Biol Blood Marrow Transplant.

